# Low-Temperature Growth of Hydrogenated Amorphous Silicon Carbide Solar Cell by Inductively Coupled Plasma Deposition Toward High Conversion Efficiency in Indoor Lighting

**DOI:** 10.1038/s41598-017-10661-y

**Published:** 2017-10-05

**Authors:** Ming-Hsuan Kao, Chang-Hong Shen, Pei-chen Yu, Wen-Hsien Huang, Yu-Lun Chueh, Jia-Min Shieh

**Affiliations:** 1 Departments of Photonics and Institute of Electro-Optical Engineering, National Chiao-Tung University, Hsinchu, 30010 Taiwan; 2 Department of Materials Science and Engineering, National Tsing Hua University, Hsinchu, 30013 Taiwan; 3 National Nano Device Laboratories, No. 26, Prosperity Road 1, Hsinchu, 30078 Taiwan

## Abstract

A p-a-SiC:H window layer was used in amorphous Si thin film solar cells to boost the conversion efficiency in an indoor lighting of 500 lx. The p-a-SiC:H window layer/p-a-Si:H buffer layer scheme moderates the abrupt band bending across the p/i interface for the enhancement of V_OC_, J_SC_ and FF in the solar spectra of short wavelengths. The optimized thickness of i-a-Si:H absorber layer is 400 nm to achieve the conversion efficiency of ~9.58% in an AM1.5 G solar spectrum. However, the optimized thickness of the absorber layer can be changed from 400 to 600 nm in the indoor lighting of 500 lx, exhibiting the maximum output power of 25.56 μW/cm^2^. Furthermore, various durability tests with excellent performance were investigated, which are significantly beneficial to harvest the indoor lights for applications in the self-powered internet of thing (IoT).

## Introduction

Self-powered Internet of Thing (IoT) chip recently has attracted much attention because the driving power of IoT chip can be harvested from environmental sources, such as indoor lighting, enabling a battery-less chip^1^. Therefore, a development of efficient energy-harvester is a key role toward the self-powered IoT chip. Among all energy-harvesters, solar cells are the most efficient energy harvesting devices, which generate the highest output powers of over 10 mA/cm^2^ and 100 μA/cm^2^ in outdoor and indoor environments, compared with other energy-harvesting devices, such as thermoelectric and piezoelectric devices^1–3^. For the indoor lights, compact fluorescent lamp (CFL) and light emitting diode (LED), which emit wavelengths of lighting from 300 to 800 nm with different intensities ranging from 400 to 1000 lx, are common lighting sources used in the world. Therefore, the selection of materials as a solar cell to harvest indoor lights plays a key role.

Up to date, dye-sensitized solar cell (DSSC), perovskite solar cell and hydrogenated amorphous silicon (a-Si:H) thin film solar cell, which have all light absorption windows of 300 nm to 800 nm, are commonly used for harvesting of indoor lighting with the best energy conversion efficiency. However, a major concern of DSSC is the use of the liquid electrolyte, containing volatile organic solvents, which is not very stable because the electrolyte will be frozen or expanded at a low or high temperature, leading to a physical damage^4^. In addition, toxic chemical components containing lead (Pb), which is quite unstable in harsh environments such as a high-temperature condition, make perovskite solar cells still far away from the industrial applications^5^. Therefore, the intrinsic hydrogenated amorphous silicon (i-a-Si:H) absorber layer with a wide band-gap behavior, which enables the strong absorption at short wavelengths in a solar spectrum with the high built-in potential between the p/n junction, is the best material choice as the solar cell for the energy-harvester of the indoor lighting. To further enhance the energy conversion efficiency of the indoor lighting, a p-type hydrogenated amorphous silicon combined with carbide, namely SiC (p-a-SiC:H), is a promising window layer to increase the light absorption at short wavelengths ranging from 2.0 to 2.5 eV^6^. Although the incorporation of carbon atoms into the p-a-Si:H film has been demonstrated to increases the V_OC_, the insufficient concentrations of carbon doped in the p-a-SiC:H film may result in a narrow band-gap, leading to the less light coupling into the absorber layer^7^. However, the excessive amount of carbon atoms in the p-a-SiC:H layer will further increase defects in the p-a-SiC:H layer and at the interface of the p-a-SiC:H/i-a-Si:H, impeding the growth of the low defect-density i-a-Si:H absorber layer with the poor collection of photo-generated carriers^8^. In addition, an abrupt or discontinuous band bending occurred at the interface of the p-a-SiC:H/i-a-Si:H can result in the degradation of fill factor (FF)^8–10^. Therefore, the p-a-SiC:H layer with the low defect density and the optimized band-gap alignment enables the efficient energy harvesting of the indoor lighting. Inductively coupled plasma chemical vapor deposition (ICP-CVD) has been demonstrated for the growth of the a-Si:H thin films with the low defect density, leading to a high conversion efficiency with the improved light-soaking stability and the less thermal stress^11,12^.

In this regard, we demonstrate the growth of the p-a-SiC:H solar cell with the optimized carbon concentration in the p-a-SiC:H window layer and the low defect density of 2.56 × 10^15^ cm^−3^ in the i-a-Si:H absorber layer. The p-a-Si:H buffer layer provides graded-band profiling and acts as the diffusion barrier to stop the diffusion of carbon atoms into the following deposited i-a-Si:H layer with low defects, exhibiting the improved J_SC_ and V_OC_, yielding a high output conversion efficiency and powers of 9.58% and 25.56 μW/cm^2^ under the AM1.5 G solar spectrum and the indoor lighting of 500 lx, respectively. The effect of the thicknesses of i-a-Si:H layers on cell performance under an AM1.5 G solar spectrum and indoor lighting has also been investigated in detail. The decrease in the fill factor under the indoor lighting is less prominent than that under the an AM1.5 G solar spectrum once the thickness of the i-a-Si:H layer increases because degradation of electric field influenced by carriers and recombination via dangling bonds are less pronounced under the indoor lighting because of a low intensity. Consequently, the ideal thickness of the i-a-Si:H absorber layer increases from 400 to 600 nm to achieve the maximum output power of 25.56 μW/cm^2^ under the indoor lighting of 500 lx without any light-soaking degradation.

## Results and Discussion

**Figure 1:**
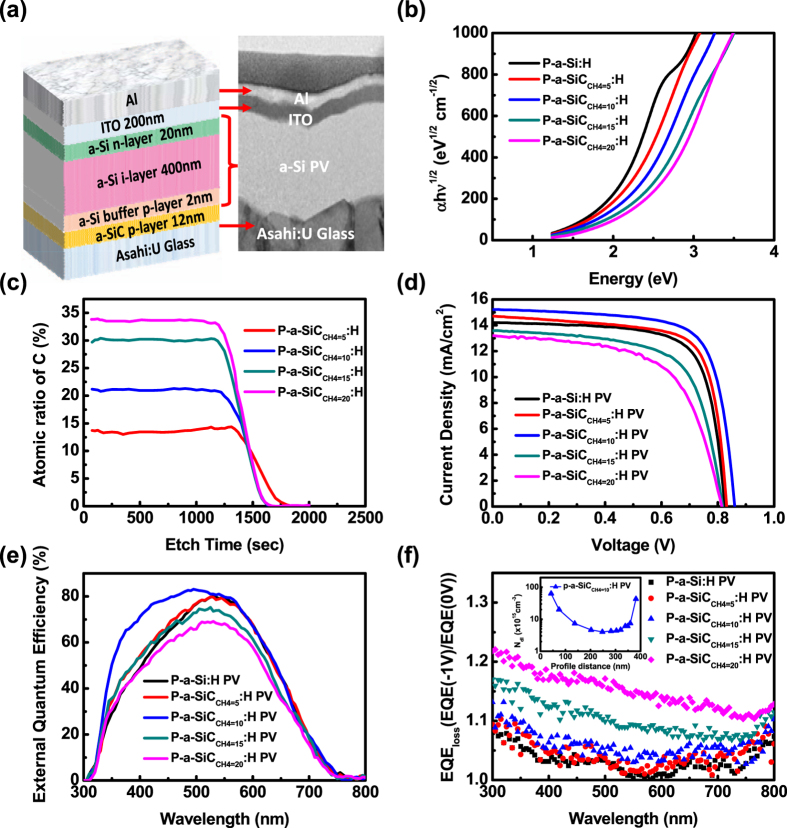
(**a**) A schematic of solar cell configuration and TEM image of a-Si:H thin film solar cells with a p-a-SiC:H window layer (abbreviated p-a-SiC:H solar cell). (**b**) Tauc’s plots and (**c**) the depth profiles of atomic ratio between C and Si for p-a-SiC:H window layers with various CH_4_ flow rates. (**d**) I-V characteristics of p-a-Si:H and p-a-SiC:H solar cells under the AM1.5 G solar spectrum (**e**) EQE and (**f**) Normalized EQE_loss_ (QE(−1 V)/QE(0 V)) of p-a-Si:H and p-a-SiC:H solar cells, respectively. (Inset shows the depth profile of bulk-defect densities in the i-a-Si:H layer retrieved from DLCP measurements for p-a-SiC_CH4 = 10_:H PV).

Figure 1(a) shows a schematic of the solar cell structure and the corresponding TEM image of a *pin*-type solar cell with a structure of Asahi substrate/p-a-SiC:H window layer (10 nm)/p-a-Si:H buffer layer (2 nm)/i-a-Si:H (400 nm)/n-a-Si:H layer (20 nm)/indium-tin-oxide (200 nm)/Al (150 nm) and area of 0.25 cm^2^. To shed light on the controllability of bandgap engineering of a-SiC:H layers, Tauc plots on p-a-Si:H and p-a-SiC:H layers as a function of gas precursor rates (CH_4_) from 5 to 20 sccm were plotted from absorption spectra as shown in Fig. 1(b). Clearly, a bandgap of 1.7 eV of the p-a-Si:H layer was confirmed while the bandgaps of p-a-SiC:H layers are controllable and increased from 2.01 to 2.28 eV due to an increase in the carbon concentration as the gas flow rate of CH_4_ increases. Furthermore, X-ray photoelectron spectroscopy (XPS) depth profiles reveal that the C/Si ratios increase from 13 to 34% as the gas flow rates of CH_4_ increase from 5 to 20 sccm as shown in Fig. 1(c). The large bandgap of the p-a-SiC:H exhibits a better absorption ability at the short wavelengths of the solar spectrum. Furthermore, we measured the I-V characteristics of the p-a-SiC:H-based solar cells integrated with various types of window layers illuminated under the AM1.5 G solar spectrum as shown in Fig. 1(d) where the a-Si:H solar cell without the p-a-SiC:H layer was used as a reference cell, with which open circuit voltage (V_OC_), short circuit current, fill factor (FF) and conversion efficiency (η) were listed in Table 1. Note that a 150-nm-thick indium tin oxide (ITO) and a 100-nm-thick Al metal layers were deposited on the a-Si:H p-i-n multilayer as the back-electrode. The p-a-Si:H cell shows a V_OC_ of 0.82 V, a J_SC_ of 14.21 mA/cm^2^, yielding a conversion efficiency of 8.51% with a FF of 73.0%. When the p-a-Si:H layer was replaced by the p-a-SiC:H window layer, V_OC_ increases from ~0.83 to ~0.86 V as the gas flow rate of CH_4_ increases from 5 to 10 sccm, which can be attributed to the higher built-in potential resulted from the enlarged bandgap of the p-a-SiC:H layer. Thanks to the increased photons with short wavelengths penetrating into the i-a-Si:H absorber layer through the p-a-SiC:H window layer as compared with the p-a-Si:H window layer, the J_SC_ raises from 14.41 mA/cm^2^ at the CH_4_ = 5 sccm to 15.45 mA/cm^2^ at the CH_4_ = 10 sccm while the FF only shows a slight decay between p-a-Si:H and p-a-SiC_CH4 = 10_:H solar cells where the p-a-SiC_CH4 = 10_:H denotes as the deposition of the p-a-SiC:H layer at the CH_4_ flow rate of 10 sccm since the p-a-Si:H buffer layer moderates the discontinuous and abrupt band bending.^18^ Furthermore, the p-a-SiC:H window layer prevents an internal diffusion of carbon atoms into the i-a-Si:H absorber layer, which leads to the low defective density in the i-a-Si:H absorber layer after the ICP-CVD, resulting in a beneficial for the collection of photo-generated carriers. Thus, the p-a-SiC_CH4 = 10_:H solar cell has the highest conversion efficiency of ~9.58%. However, the further increase in carbon concentrations leads to degraded performance, especially for the p-a-SiC_CH4 = 20_:H solar cell, which suffers from low V_OC_ (0.81 V), J_SC_ (12.42 mA/cm^2^) and FF (61.1%), yielding a poor conversion efficiency of ~6.15%. The reason can be explained that the excess carbon atoms in the p-a-SiC_CH4 = 20_:H window layer diffuse into the i-a-Si:H absorber layer with sufficiently high interfacial defects, leading to the high recombination of the photo-generated carriers in the i-a-Si:H absorber layer. Figure 1(e) shows the external quantum efficiency (EQE) of all cells, including the a-Si:H and p-a-SiC:H solar cells at different gas flow rates of CH_4_, respectively. Compared to the p-a-Si:H solar cell, the collection efficiency of p-a-SiC_CH4 = 10_:H solar cell shows the dramatically enhancement at short wavelengths of the solar spectra while the EQE decreases at the gas flow rate of CH_4_ over 10 sccm. To further understand the photo-generated carriers trapped across the interface of p-a-SiC/i-a-Si:H layers and inside the i-a-Si:H absorber layer, the reverse bias quantum efficiency was measured where a negative electric field enables trapped carriers across the interface as shown in Fig. 1(f) where the EQE_loss_ is defined by the ratios of EQE at a negative bias of −1 V and without bias (0 V) at different wavelengths. Obviously, the p-a-SiC_CH4 = 10_:H window layer shows the EQE_loss_ < 1.1 in the blue light region, which is comparable with the p-a-Si:H layer, suggesting the fairly low defects across the interface of p-a-SiC/i-a-Si:H layers and inside the i-a-Si:H layer. However, the EQE_loss_ becomes severe when the gas flow rate of CH_4_ exceeding 10 sccm. This is due to high carbon concentration is prone to diffuse into the i-a-Si:H absorber layer, forming as the recombination centers. The higher EQE_loss_ of the p-a-SiC_CH4 = 20_:H solar cell indicates the severe carbon diffusion from the p-a-SiC:H window layer to the i-a-Si:H absorber layer, which is consistent with degraded results found in EQE measurements (Fig. 1e). To confirm the film quality of the i-a-Si:H absorber layer, we determined the integrated defect density of the p-a-SiC_CH4 = 10_:H solar cell as a function of the depth from the top of the p-i-n active layer by drive-level capacitance profiling (DLCP) measurement as shown in inset of Fig. 1(f). The p-a-SiC_CH4 = 10_:H solar cell exhibits the extremely low bulk-defect density of 2.56×10^15^ cm^−3^ in the i-a-Si:H layer, which is comparable with our previous work^12^.

**Table 1:** Performances of p-a-Si:H, p-a-SiC_CH4 = 5_:H, p-a-SiC_CH4 = 10_:H, p-a-SiC_CH4 = 15_:H and p-a-SiC_CH4 = 20_:H solar cells under the AM1.5 G solar spectrum where the cell area of 0.25 cm^2^ was used.

Illumination condition	Cell type	V_OC_ (volt)	J_SC_ (mA/cm^2^)	FF (%)	Efficiency (%)
AM1.5 G solar spectrum	P-a-Si:H	0.82	14.21	73.0	8.51
	P-a-SiC_CH4 = 5_:H	0.83	14.41	72.5	8.67
	P-a-SiC_CH4 = 10_:H	0.86	15.45	72.1	9.58
	P-a-SiC_CH4 = 15_:H	0.82	13.58	66.0	7.35
	P-a-SiC_CH4 = 20_:H	0.81	12.42	61.1	6.15

**Figure 2:**
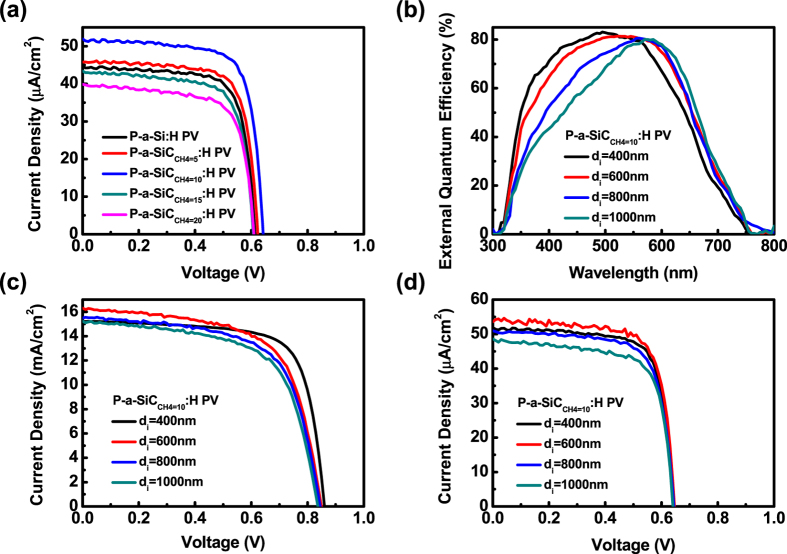
(**a**) I-V characteristics of p-a-Si:H and p-a-SiC:H solar cells under the indoor lighting of 500 lx (white FCL illumination). (**b**) EQE of p-a-SiC_CH4 = 10_:H solar cells as a function of the thickness of absorber intrinsic layers (d_i_). (**c**) and (**d**) I-V characteristics of the p-a-SiC_CH4 = 10_:H solar cell as a function of d_i_ under the AM1.5 G solar spectrum and the indoor lighting of 500 lx (white FCL illumination).

To investigate electrical behaviors of a-Si:H solar cells under the indoor lighting environments, the I-V characteristics of all solar cells illuminated by the indoor lighting of 500 lx, which is approximately of 162 μW/cm^2^, were measured as shown in Fig. 2(a) and the corresponding V_OC_, Jsc, FF and conversion efficiency in term of output power in an unit of µW/cm^2^ were tabulated in Table 2. Clearly, all solar cells show the reduced V_OC_ and J_SC_ but the enhanced FF under the indoor lighting of 500 lx compared with that illuminated under an AM1.5 G solar spectrum. The enhanced FF can be explained due to the less light-induced metastable defects by the weak intensity of the indoor lighting. The enhanced FF is contributed not only the less recombination centers from charged dangling bonds but also the less field deformation from trapped charges, which are proportional to free carriers under the low level of the indoor lighting. Conversely, a decrease in J_SC_ results from the low intensity of lighting, accompanying with the reduction of V_OC_ given by

$${V}_{oc}=(\frac{\eta kT}{q})\mathrm{ln}(\frac{{J}_{sc}}{{J}_{s}}+1)$$

Where η, k, T and J_S_ represent the ideal factor, the Boltzmann constant, the absolute temperature and the saturated current, respectively. It is beneficial for utilizing the indoor lighting sources by introducing a wide band-gap p-SiC:H window layer to improve the J_SC_ and V_OC_. Accordingly, compared cell performance with the a-Si:H solar cell, the p-a-SiC_CH4 = 10_:H solar cell shows a V_OC_ of 0.64 V, a J_SC_ of 51.90 μA/cm^2^, a FF of 74.9%, and an output power of 24.88 μW/cm^2^. Furthermore, the different thicknesses of i-a-Si:H absorber layers from 400 to 1000 nm were also optimized in order to reduce the formation of light-induced defects in the i-a-Si:H layers under the indoor lighting of 500 lx. Figure 2(b) shows the dependence of EQE spectra of the p-a-SiC_CH4 = 10_:H solar cell with different i-a-Si:H thicknesses (d_i_) from 400 to 1000 nm. The EQE responses shift toward a long wavelength region as the d_i_ increases. Figure 2(c) and (d) show I-V characteristics of the p-a-SiC_CH4 = 10_:H solar cell illuminated by the AM1.5 G solar spectrum and the indoor lighting of 500 lx, respectively. The corresponding V_OC_, J_SC_, FF and conversion efficiency with different d_i_ were tabulated in Tables 3 and 4, respectively. Under the AM1.5 G solar spectrum, the J_SC_ increases as the d_i_ increases because of the improved absorption behaviors of photons in the i-a-Si:H absorber layer, achieving the maximum J_SC_ in the 600 nm-thick i-a-Si:H solar cell. In contrast, the J_SC_ and the V_OC_ decrease while the d_i_ exceeds the thickness of 600 nm. This is because that the reduced V_OC_ separates less photo-generated carriers, resulting in the decrease in J_SC_. In addition, the thick i-a-Si:H layer further induces more bulk-defects to reduce the parallel resistance, leading to poor FF. As a result, the optimized thickness of the i-a-SiC:H layer under the AM1.5 G solar spectrum to be at 400 nm. For the performance of solar cells under the indoor lighting of 500 lx, no significant variation tendency at V_OC_ was found while the FF slightly decreases from 74.9 to 70.1% as d_i_ increases from 400 to 1000 nm, which is most likely attributed to less recombination centers and weak deformation caused by the internal electric field (Fig. 2d). High parallel resistance results from the less light-induced metastable defects, further extending the lifetime of solar cells applied in the indoor lighting environment. Consequently, the optimized thickness of the i-a-SiC:H film was found to be 600 nm with the highest V_OC_ of 0.64 V, J_SC_ of 54.18 μA/cm^2^ and FF of 73.7%, yielding a maximum output power of 25.56 μW/cm^2^, respectively.

**Table 2:** Performances of p-a-Si:H, p-a-SiC_CH4 = 5_:H, p-a-SiC_CH4 = 10_:H, p-a-SiC_CH4 = 15_:H and p-a-SiC_CH4 = 20_:H solar cells under the indoor lighting of 500 lx by white FCL.

Illumination condition	Cell type	V_OC_ (volt)	J_SC_ (μA/cm^2^)	FF (%)	Output power (μW/cm^2^)
500 lx white FCL	P-a-Si:H	0.61	44.81	75.2	20.56
	P-a-SiC_CH4 = 5_	0.62	45.81	75.1	21.33
	P-a-SiC_CH4 = 10_	0.64	51.90	74.9	24.88
	P-a-SiC_CH4 = 15_	0.61	43.65	72.6	19.33
	P-a-SiC_CH4 = 20_	0.60	39.86	70.5	16.86

**Table 3:** Performances of the p-a-SiC_CH4 = 10_:H solar cell with different di thicknesses of 400, 600, 800 and 1000 nm under the AM1.5 G solar spectrum, respectively.

Illumination condition	di thickness	V_OC_ (volt)	J_SC_ (μA/cm^2^)	FF (%)	Output power (μW/cm^2^)
AM1.5 G solar spectrum	400 nm	0.85	15.45	72.1	9.50
	600 nm	0.85	16.19	68.2	9.40
	800 nm	0.85	15.55	63.8	8.39
	1000 nm	0.84	15.10	63.1	7.98

**Table 4:** Performances of the p-a-SiC_CH4 = 10_:H solar cells with different d_i_ thicknesses of 400, 600, 800 and 1000 nm under the indoor lighting of 500 lx by the white FCL, respectively.

Illumination condition	di thickness	V_OC_ (volt)	J_SC_ (μA/cm^2^)	FF (%)	Output power (μW/cm^2^)
500 lx white FCL	400 nm	0.64	51.9	74.9	24.88
	600 nm	0.64	54.18	73.7	25.56
	800 nm	0.64	50.97	71.5	23.37
	1000 nm	0.64	48.6	70.1	21.84

**Figure 3:**
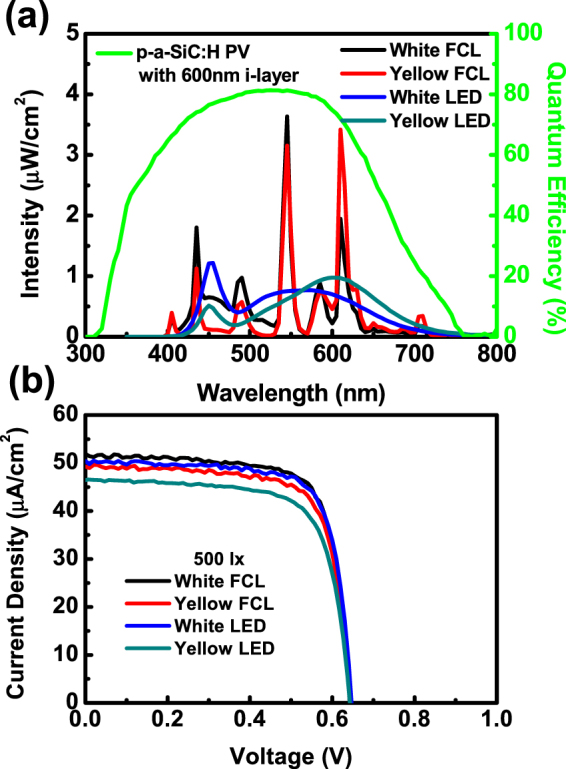
(**a**) Spectra of white/yellow FCL/LED lights with the lighting of 500 lx. (**b**) I-V characteristics of the p-a-SiC_CH4 = 10_:H solar cell with the d_i_ of 600 nm under white/yellow FCL/LED lights with the lighting of 500 lx.

To further shed light on the I-V behaviors of a-SiC:H solar cells under the indoor lights, EQE spectra of the p-a-SiC_CH4 = 10_:H solar cell with the optimized i-a-Si:H layer thickness of 600 nm under different indoor lights, including white/yellow compact fluorescent lamps (CFL) and light emitting diode (LED), were measured. The corresponding I-V characteristics are shown in Fig. 3(a) and (b) where V_OC_, J_SC_, FF and output power were tabulated in Table 5, respectively. It is obvious that the spectra of all indoor lights are almost located at wavelengths of 400–700 nm and matches well with the EQE response of the a-Si:H PV cell. Especially, the spectra of white FCL and LED show strong intensities at short wavelengths of 400–500 nm, which can be absorbed by the i-a-Si:H layer by incorporation of the p-a-SiC_CH4 = 10sccm_:H layer. The spectra of yellow FCL and LED exhibit prominent intensity at the long wavelengths of 550–700 nm, which can be absorbed by the 600-nm-thick i-a-Si:H absorbed layer. It is demonstrated that the optimized PV cell can provide the output power ranging from 22.11 to 25.56 μW/cm^2^ under white/yellow FCL/LED, implying that this optimized structure is well suitable for the harvesting of the indoor lighting.

**Table 5:** Performances of the p-a-SiC_CH4 = 10_:H solar cell with the d_i_ thickness of 600 nm under the indoor lighting of 500 lx by white/yellow FCL/LED.

Illumination condition	d_i_ thickness	V_OC_ (volt)	J_SC_ (μA/cm^2^)	FF (%)	Output power (μW/cm^2^)
500 lx white FCL	600 nm	0.64	54.18	73.7	25.56
500 lx yellow FCL	600 nm	0.64	51.43	73.8	24.29
500 lx white LED	600 nm	0.64	52.36	73.7	24.70
500 lx yellow LED	600 nm	0.64	48.60	73.6	22.89

**Figure 4:**
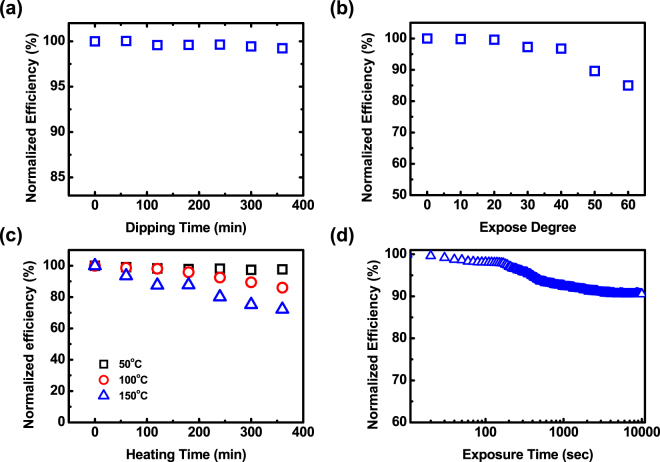
(**a**) Water resistant tests, (**b**) angle-dependent and (**c**) heating tolerance measurements of the p-a-SiC_CH4 = 10_:H solar cell with the d_i_ of 600 nm. (**d**) The conversion efficiency degradation of p-a-SiC_CH4 = 10_:H PV with the d_i_ of 600 nm as a function of exposure time under six AM1.5 G solar spectra at 60 °C.

To qualify the stability and lifetime in a real application, the p-a-SiC_CH4 = 10_:H solar cell with the 600 nm-thick-i-a-Si:H absorber layer is subject to various durability tests. Figure 4(a) shows the normalized efficiency measurements under a water-resistant test as a function of the dipping time. The conversion efficiency remains to be unchanged after 360 mins in the water, demonstrating an excellent water-resistant capability. Figure 4(b) shows the angle-dependent measurements under the indoor lighting of 500 lx. Interestingly, the conversion efficiency is not significantly changed by the incident angle of >40° where a ~5% degradation of the conversion efficiency was found. The ~15% degradation of the conversion efficiency occurs once the incident angle researches into 60°, which promises the wide-angle utilization of the incident lighting in the real practice. Figure 4(c) shows the thermal stress testes as a function of heating time at different heating temperatures of 50 to 150 °C. A decrease by only ~28% after the thermal stress of 360 mins at the heating temperature of 150 °C was confirmed, clearly demonstrating the highly stable a-SiC:H-based solar cell operated in a high-temperature environment. Furthermore, an endurance test under the continuous exposure of six AM1.5 G solar spectra at a heating temperature of 60 °C was conducted as shown in Fig. 4(d). The stabilized efficiency decreases by ~8.2% after the exposure of 10000 seconds. Such excellent performance can be explained by the excellent quality of the i-a-Si:H layer due to low defects in the p-a-SiC:H layer and the interface of the p-a-Si:H/i-a-Si:H layers where the p-a-Si:H layer acted as a buffer layer, eliminating carbon diffusion through the interface. The result is fairly consistent with the bias EQE measurements. Note that a solar cell with better light-soaking stability under the AM1.5 G solar spectrum responds to the lifetime of solar cell illuminated by the fluorescence lamp.

## Conclusions

The introduce of p-a-SiC:H layer in the a-Si:H solar cell shows a high conversion efficiency of 9.58% and 25.56 uW/cm^2^ under 1-Sun and 500 lx illumination, which results from the continuous band bending and low defect density of i-a-Si:H layer. When the ideal thickness of i-a-Si:H layer was changed from 400 to 600 nm, no light-soaking can be observed under the 500 lx illumination. The findings here provides a path for harvesting the indoor light for real application. The optimized solar cell with the light harvesting scheme at short wavelengths and high stability is well suited for commonly used future applications in self-powered IoTs under the indoor environment.

## Methods

### ICP CVD growth of A p-a-SiC:H and fabrication process Solar Cell

For the fabrication of p-a-Si:H and p-a-SiC:H PV, the a-Si:H p-i-n multilayer with p-a-Si:H window layer (12 nm)/i-a-Si:H layer (400 nm)/n-a-Si:H layer (20 nm) and p-a-SiC:H window layer (10 nm)/p-a-Si:H buffer layer (2 nm)/i-a-Si:H layer (400–1000 nm)/n-a-Si:H layer (20 nm) were deposited on the Asahi substrates (SnO_2_:F/glass) by 13.56 MHz inductively coupled plasma chemical CVD (ICP-CVD) with different plasma power densities of 30~100 mW/cm^2^, which can produce a-Si:H thin films with low defects at a substrate temperature of 200 °C. For the p-a-SiC:H window layer, the gas flow rate of CH_4_ were varied from 5 to 20 sccm whereas the flow rate of Si_2_H_6_ and B_2_H_6_ were kept at 7 and 10 sccm, respectively. For the p-a-Si:H window layer, the flow rate of CH_4_ was kept at 0 sccm, whereas the flow rate of Si_2_H_6_ and B_2_H_6_ were remained the same as stated before. To minimize the defects at the p-a-SiC:H/i-a-Si:H interface, we have introduced a buffer layer between the p-a-SiC:H and the i-a-Si:H layers by grading the gas flow rate of CH_4_ from an initial value down to zero. The concentration of hydrogen inclusion and the microstructure parameter of the i-a-Si:H films were deduced from the integrated peak area of the 630 cm^−1^ peak and ratio of the spectral peaks I_2070_/(I_2070_ + I_2000_) of Fourier-transform infrared (FTIR) absorption spectra. Here, I_2070_ and I_2000_ denote the integrated infrared absorption peak areas of stretching mode of Si-H bonds, locating at internal interfaces (2070 cm^−1^) and of the isolated Si-H bonds (2000 cm^−1^), respectively. We estimated the hydrogen content and microstructure parameter of the i-a-Si:H films to be about 10.1% and 0.08, implying a dense lattice network with a very low level of voids^13^. Afterward, reflective contacts of 150-nm-thick indium-tin-oxide (ITO) and a 100-nm-thick-Al metal layers were then deposited by direct current sputtering on the back side. The ITO layers have high electrical conductivity (<1 × 10^–3^ Ωcm) and optical transmittance (>90%, 400–900 nm), which significantly improve the light collection and reduce the series resistance of the devices.

### Characterizations

I-V characteristics were measured under an AM1.5 G global sun simulator (Oriel Sol3A) with the light intensity of 1000 W/m^2^ irradiance. Fluorescent tubes and LED tubes with daylight white and warm white spectral distribution were the most common types of indoor lights. The measurement setup includes two fluorescent tubes (Philip TL5 14 W/6500 K and 14 W/3000 K) and two LED tubes (Everlight LED 10 W/6500 K and 10 W/3000 K). The illuminance of fluorescents tube and LED tubes were fixed at recommended office lighting levels of 500 lx by adjusting the distance between light source and PV devices. The illuminance and spectral distribution of different lights were calibrated with lux meter and spectroradiometer. The light-soaking measurements were performed under the light irradiance of 6000 W/m^2^ (six AM1.5 G spectra). The device reached accordingly a steady-state temperature at 60 °C due to the irradiation, thereby accelerating photo-degradation of the device. High-resolved transmission electron microscopy (HR-TEM) images obtained by JEOL JEM-3000F FE-TEM with an accelerated bias of 300 kV with a point to point resolution of 0.17 nm was used to observe the device configuration. The p-a-SiC:H films were also deposited on corning 7059 glass and silicon substrates for measurements of n&k analyzer and X-ray photoelectron spectroscopy (XPS). The bandgap was determined by Tauc’s bandgap (extrapolation of (αE)^1/2^) from the evaluation of visible/UV transmission and reflection spectra.
